# Structural and functional insights into the G protein-coupled receptors: CB1 and CB2

**DOI:** 10.1042/BST20221316

**Published:** 2023-08-10

**Authors:** Christina A. Brust, Matthew A. Swanson, Laura M. Bohn

**Affiliations:** 1 Department of Molecular Medicine, The Herbert Wertheim UF Scripps Institute for Biomedical Innovation and Technology, Jupiter, FL 33458, U.S.A.; 2 The Skaggs Graduate School of Chemical and Biological Sciences at Scripps Research, La Jolla, CA 92037, U.S.A.

**Keywords:** cannabinoids, cryo-electron microscopy, crystallography, G-protein-coupled receptors

## Abstract

The cannabinoid receptors CB1 and CB2 mediate a variety of physiological processes and continue to be explored as desirable drug targets. Both receptors are activated by the endogenous endocannabinoids and the psychoactive components of marijuana. Over the years, many efforts have been made to make selective ligands; however, the high degree of homology between cannabinoid receptor subtypes introduces challenges in studying either receptor in isolation. Recent advancements in structure biology have resulted in a surge of high-resolution structures, enriching our knowledge and understanding of receptor structure and function. In this review, of recent cannabinoid receptor structures, key features of the inactive and active state CB1 and CB2 are presented. These structures will provide additional insight into the modulation and signaling mechanism of cannabinoid receptors CB1 and CB2 and aid in the development of future therapeutics.

## Introduction

**Figure 1.**
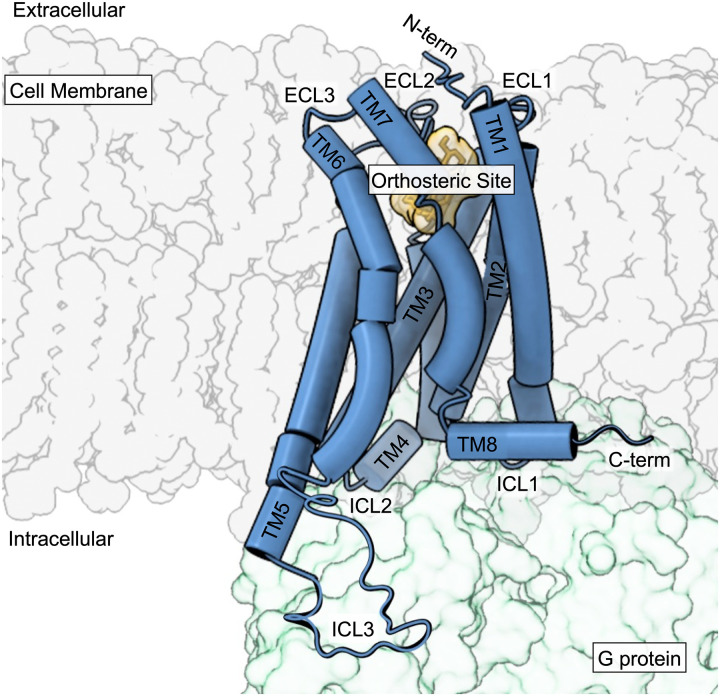
Class A GPCR general structure. A ligand-bound GPCR interacting with the heterotrimeric G protein.

Human endocannabinoid signaling is primarily mediated by the class A G protein-coupled receptors (GPCRs) CB1 and CB2. Class A GPCRs consist of an extracellular N-terminal domain, seven transmembrane spanning alpha helices, three extracellular loops, three intracellular loops, and an intracellular G protein binding domain (Figure 1). Heterotrimeric G proteins consist of α, β, and γ subunits. Following receptor activation, GTP is exchanged for GDP at the α subunit, activating it, resulting in its dissociation from the βγ subunits, initiating downstream signaling. While CB1 and CB2 primarily couple to inhibitory G_αi/o_ proteins [1], they are also known to interact with stimulatory G proteins such as G_αs_ and G_αq/11_ [2]. These interactions are largely dependent on the cell or tissue type. GPCR activation typically results in receptor phosphorylation by G protein receptor kinases (GRKs) and subsequently the recruitment of βarrestins. CB1 and CB2 receptor desensitization and internalization is mediated by the recruitment of βarrestin1 and 2 [3,4].

CB1, initially identified in rats in 1988 [5], is the most abundantly expressed GPCR in the brain and predominately localized in the central nervous system (CNS) [6]. CB1 is also peripherally expressed in the liver, reproductive tissues, gastrointestinal tract, cardiovascular system, and skeletal muscle [7]. CB2 was discovered in 1993 and was coined as the ‘peripheral cannabinoid receptor', since it originally went undetected in the CNS [8]. However, while CB2 is predominantly expressed in immune cells, it is also present in the CNS to a lesser extent [9,10]. CB1 and CB2 share 44% overall sequence homology and 68% homology across the transmembrane domain. Sequential differences between the two receptors are largely at the N-terminus, extracellular loop II (ECL2), and C-terminal domain [11].

**Figure 2.**
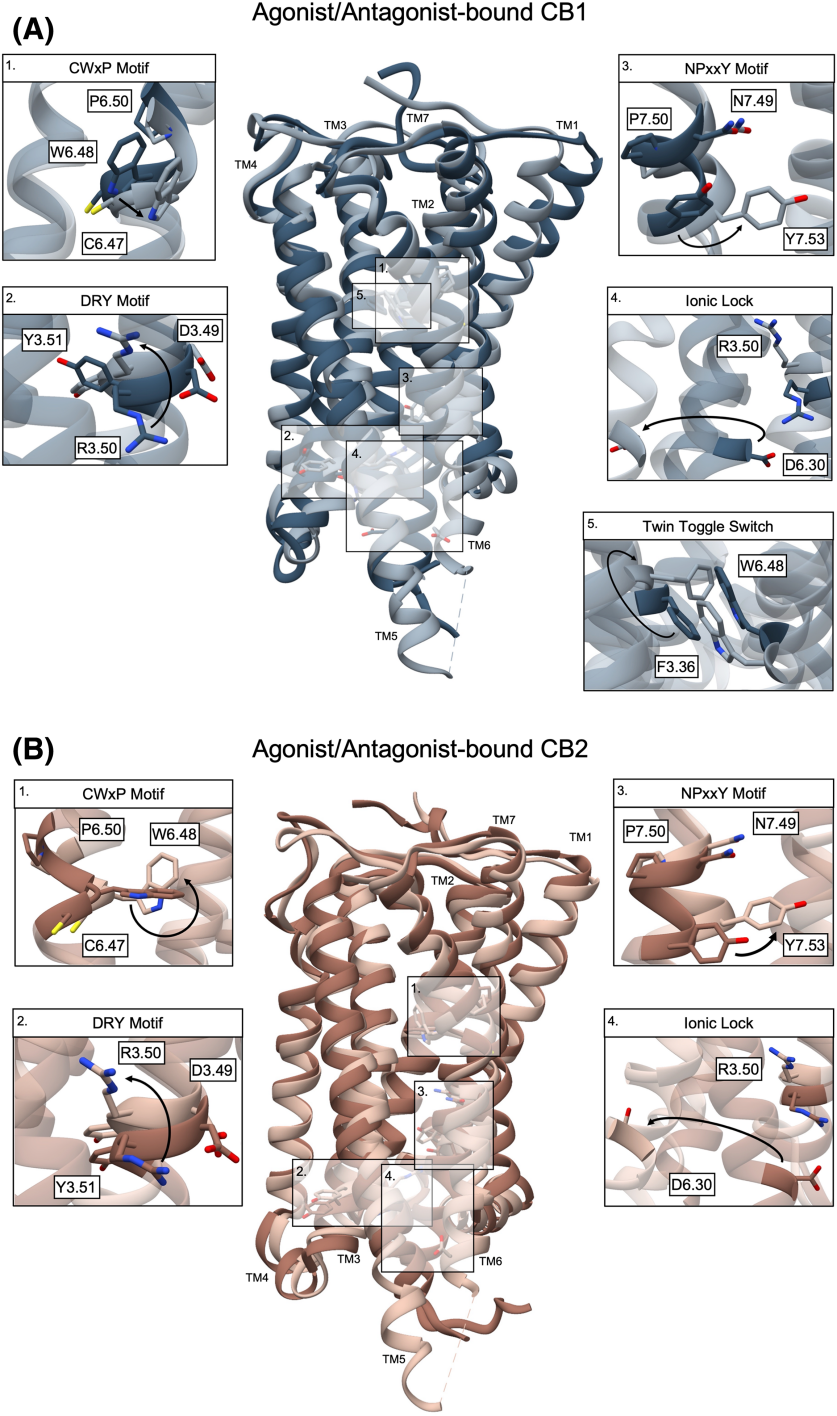
Comparing the confirmational changes of key molecular switches between the antagonist- and agonist-bound CB1 and CB2. (**A**) Antagonist-bound CB1 receptor (PDB: 5TGZ) in dark blue with the agonist-bound CB1 (PDB: 6KPG) in grey. (**B**) Antagonist-bound CB2 receptor (PDB: 5ZTY) in brown, overlayed with the agonist-bound CB2 structure (PDB: 6KPF) in beige. Arrows within each panel indicate shifts from the inactive to active state receptor.

Receptor activation is characterized by the rotation and outward movement of the intracellular end of transmembrane helix 6 (TM6), increasing the distance between TM3 and TM6, and allowing effector proteins to bind. A few of the most commonly accepted molecular switches include the ionic lock and E/DRY motif, the tryptophan toggle switch (CWxP), and the tyrosine toggle switch (NPxxY) (Figure 2; Table 1) [12]. Residues in class A GPCRs are denoted using Ballesteros–Weinstein numbering, where the first number designates the transmembrane helix, and the second is the residue's location relative to the most conserved residue within that transmembrane helix [13]. For example, R3.50 of the ionic lock is located on TM3, and as it is the most conserved residue on TM3, it is followed by X.50.

**Table 1:** Conserved molecular switches of class A GPCRs

	Location	CB1	CB2
Ionic Lock	R3.50/D6.30	R214/D338	R131/D240
E/DRY motif	D3.49, R3.50, Y3.51	D213, R214, Y215	D130, R131, Y132
Tryptophan toggle switch (CWxP)	W6.48	W356	W258
Twin toggle switch	W6.48/F3.36	W356/F200	Absent
Tyrosine toggle switch (NPXXY)	Y7.53	Y397	Y299

Residues are indicated for the human cannabinoid receptors CB1 and CB2.

CB1 agonists may have potential as a treatment for pain and inflammation [14], Multiple sclerosis [15,16], and neurodegenerative disorders [17]. While CB1 antagonists have been used to treat obesity-related metabolic disorders [18], mental illnesses [19,20], liver fibrosis [21], and nicotine addiction [22]. CB2 agonists have been pursued for the treating inflammatory and neuropathic pain, neuroinflammation, and neurodegenerative disorders as well as immunomodulation [23–25]. Due to the therapeutic potential associated with endocannabinoid system, the development of novel ligands targeting CB1 and CB2 remains a promising field. However, the majority of cannabinoid receptor ligands display some affinity at both CB1 and CB2, therefore studying either receptor in isolation is challenging. Continuing to acquire insight into cannabinoid receptor structure and function will ultimately aid in the development of future therapeutics.

## Structural determination of cannabinoid receptors

### Antagonist-bound CB1

In 2016 the first crystal structure of the antagonist-bound CB1 receptor was solved (PDB: 5TGZ) [26]. The 2.8 Å structure of CB1 bound to AM6538 depicted the ligand residing low within the binding pocket, located directly above W356^6.48^, the conserved toggle switch and formed hydrophobic contacts with ECL2 and the N-terminus. While the N-terminus is truncated, residues 99–112 form a V-shaped loop that inserts into the ligand-binding pocket. The pyrazole ring core formed hydrophobic interactions with F170^2.57^, F379^7.35^, and S383^7.39^ and was capped by M103^N-term^. Arm 1 (2,4-dichlorophenyl ring) formed edge-face π–π interactions with the sidechain of F170^2.57^ and the backbone amide between G166^2.53^ and S167^2.54^. The substituted ring moiety forms hydrophobic interactions with V196^3.32^, W356^6.48^, C386^7.42^, L387^7.43^, and M103^N-term^. The phenyl ring of Arm 2 (4-aliphatic chain substituted phenyl ring) forms π–π interactions with F102^N-term^, F268^ECL2^, W356^6.48^ and hydrophobic interactions with L193^3.29^, V196^3.32^, L359^6.51^ (Table 2). The triple bond forms π–π interactions with F268^ECL2^ and W356^6.48^ and hydrophobic interactions with L193^3.29^, V196^3.32^, T197^3.33^, L359^6.51^, and M363^6.55^. Although the nitrate moiety was not resolved in the crystal structure, interactions with T197^3.33^, Y275^5.39^, and W279^5.43^ were identified via molecular docking. Arm 3 (piperidin-1-ylcarbamoyl) forms hydrophobic interactions with M103^N-term^, I105^N-term^, I119^1.35^, S123^1.39^, F170^2.57^, F174^2.61^, A380^7.36^, S383^7.39^, and M384^7.40^. The most prevalent interactions occur around F170^2.57^, which interacts with the central pyrazole ring and arms 1 and 3. These interactions pushed F170^2.57^ toward TM1 resulting in a tilt of the last two turns (residues 170–177) of TM2 toward TM1. This pushes TM1 by ∼27 Å due to interactions between F170^2.57^ and F174^2.61^.

**Table 2:** Key residues and interactions at the ligand-binding interface organized by PDB

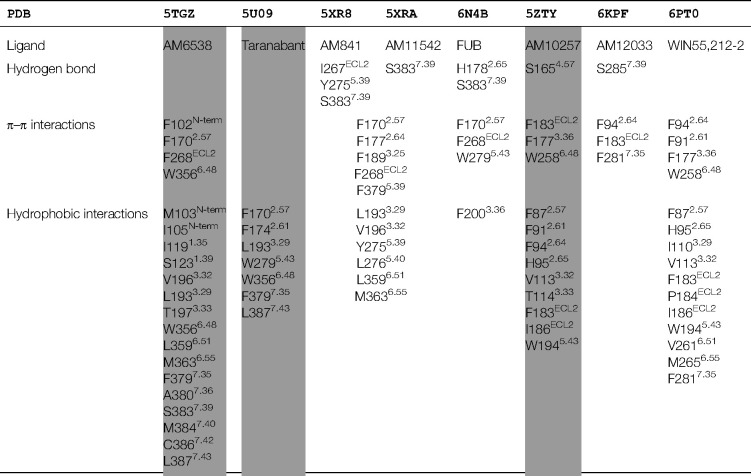

Antagonist-bound structures are highlighted in grey.

Shortly thereafter, a second crystal structure of CB1 was released, with the antagonist taranabant (PDB: 5U09) [27]. Taranabant is an analog of SR141716A, or Rimonabant [28]. This structure depicted direct contacts between taranabant and F170^2.57^, F174^2.61^, L193^3.29^, W279^5.43^, W356^6.48^, F379^7.35^, and L387^7.43^. Similar to the CB1-AM6538 structure, the truncated N-terminus was folded over the ligand-binding site, shielding taranabant from the solvent.

### Agonist-bound CB1

The first active state CB1 structures were released in 2017 with the wash-resistant, (-)-*trans*-Δ^9^-tetrahydrocannabinol (Δ^9^-THC) analogs AM11542 (PDB: 5XRA) and AM841 (PDB: 5XR8) [29]. In the AM11542-bound CB1 crystal structure, the tricyclic ring system forms π–π interactions with F177^2.64^, F268^ECL2^, F189^3.25^, and F379^7.35^, while the phenolic hydroxyl group at C1 forms a hydrogen bond with S383^7.39^. In the AM841-bound structure, the C11 hydroxyl group interacts with I267^ECL2^ and the isothiocyanate group forms a hydrogen bond with the alkyl chain of both agonists Y275^5.39^. The alkyl chain of the agonists extends into a long channel formed by TM3, TM5, and TM6, forming hydrophobic interactions with L193^3.29^, V196^3.32^, Y275^5.39^, L276^5.40^, L359^6.51^, and M363^6.55^. The C1’-gem-dimethyl group forms hydrophobic interactions with F200^3.36^, L359^6.51^, and M363^6.55^. Compared with the CB1-AM6538 structure, the extracellular part of TM1 bends inwards by ∼6.6 Å and TM2 rotates in by ∼6.8 Å. The largest change is observed by the intracellular portion of TM6 which moves outward by ∼8 Å. A ∼53% reduction in the volume of the orthosteric binding pocket is also observed upon agonist binding, indicating fluidity in the CB1 structure. Although the previous inactive state CB1 structures depicted the N-terminus occupying the ligand-binding domain, that was not resolved in the agonist-bound structure. An additional distinction between the agonist and the antagonist-bound structures is the presence of a cholesterol molecule on the surface of TM2, TM3, and TM4 in the active state CB1. This suggests the cholesterol may act as an allosteric modulator, especially since the cholesterol derivative pregnenolone is a known allosteric modulator of CB1 [30].

The CB1 twin-toggle switch, comprised of F200^3.36^ and W356^6.48^, engage in π–π interactions in the inactive conformation (Figure 1A). In the AM11542-CB1 structure, there is a rotation of TM3 and a flip of F200^3.36^ toward the ligand-binding pocket, promoting hydrophobic interaction with the C1′gem-dimethyl group of AM11542. TM6 simultaneously rotates, causing W356^6.48^ to swing outwards, and disrupting the π–π stacking of the twin-toggle switch. Additional disruptions were observed in the polar network around the E/DRY motif, the intra-helical salt bridge between D213^3.49^ and R214^3.50^, and the ionic lock between R214^3.50^ and D338^6.30^. Separation of the ionic lock results in a rotamer shift of D338^6.30^ and causes TM6 to move away from TM3. There also appears to be a partial unwinding of TM7 around W397^7.53^.

In 2019 the first cryo-EM structure of CB1 in complex with G_i_ was captured with agonist MDMB-Fubinaca (FUB) (PDB: 6N4B) [31]. FUB is a component of synthetic cannabinoid mixes such as ‘Spice' or ‘K2'. The indazole ring of FUB interacts with F200^3.36^ of the twin toggle switch and disrupts the π–π stacking network around W356^6.48^ causing it to rotate inward. This causes TM6 to straighten, creating a larger cavity for the G protein to access. It is proposed that FUB stabilizes the twin toggle due to strong aromatic interactions with F200^3.36^, resulting in an increase in efficacy compared with the partial agonist Δ^9^-THC which doesn't have a major stabilizing group. Molecular dynamics (MD) simulations from this study illustrate TM1 moving outward and creating a gap between TM1 and TM7, similar to one observed in the CB1-taranabant structure. This gap would potentially allow the ligand to transiently access the orthosteric binding site through the lipid-bilayer.

In 2020, an additional CB1-G_i_ cryo-EM structure was released with the agonist AM841 (PDB: 6KPG) [32]. This AM841-CB1-G_i_ complex is largely comparable to the 2016 AM841-CB1 crystal structure, aside from the G protein binding interface, which will be discussed in further detail separately.

Although synthetic cannabinoids continue to play a critical role in structural determination, identifying how endogenous ligands influence receptor structure and function is fundamental to our understanding of CB1 and CB2. The most recent cryo-EM structure of CB1-G_i_ was captured in complex with the endocannabinoid analog AMG315 [33]. AMG315 is an analog of N-arachidonoylethanolamide or AEA. While the acyl chain resides deep within orthosteric pocket, the carbonyl head group interacts with the residues F268^ECL2^ and I267^ECL2^. Seeing that the CB1 ECL2 is distinct from that of CB2, these interactions could explain the selectivity AMG315 exhibits for CB1 over CB2.

### Antagonist-bound CB2

The first CB2 structure was captured in complex with the antagonist AM10257 (PDB: 5ZTY) [34]. The core pyrazole ring of AM10257 forms π–π interactions with F183^ECL2^ and hydrophobic interactions with F87^2.57^ and V113^3.32^. Arm 1 (benzene ring) forms π–π interactions with F117^3.36^ and W258^6.48^. Arm 2 (5-hydroxypentyl chain) forms hydrophobic interactions with T114^3.33^, F183^ECL2^, I186^ECL2^, and W194^5.43^. The terminal hydroxy group on arm 2 is involved in a hydrogen-bonding network between a water molecule and S165^4.57^. Arm 3 (adamantyl group) forms hydrophobic interactions with F87^2.57^, F91^2.61^, F94^2.64^, H95^2.65^, and F183^ECL2^.

Contrary to CB1, CB2 does not contain a dual toggle switch. In the antagonist-bound structure, AM10257 constrains the CB2 toggle switch W258^6.48^ in a relatively rare rotamer that is only observed in muscarinic acetylcholine receptors [35–37] and two neurotensin receptors [38]. This rotamer restricts the outward movement of TM6, locking the receptor in an inactive conformation. The inactive CB2 receptor structure holds a conformation that closely resembles that of the agonist-bound CB1-AM11542 structure as the N-termini adopt a short helix before Y24^N-term^ of CB2 and F108^N-term^ of CB1. The antagonist-bound CB2 structure diverges from the antagonist-bound CB1 structure, and is more similar to other lipid receptors such as S1P1 and LPA1 bound to the antagonists ML056 [39] and ONO9780307 [40], respectively. The truncated N-terminus of CB2 does not reside within the orthosteric pocket, as previously shown in the antagonist-bound CB1 structures, and the overall volume of the ligand-binding pocket of CB1-AM6538 is ∼2 fold larger than that of the antagonist-bound CB2 and agonist-bound CB1.

### Agonist-bound CB2

In 2020, the first active state CB2 structures were captured in complex with G_i_. Hua et al. [32] reported a crystal structure of CB2-G_i_ bound to the agonist AM12033 (PDB: 6KPC), alongside two cryo-EM structures, one of AM12033-CB2-G_i_ (PDB: 6KPF) and a second with AM841-CB1-G_i_ (PDB: 6KPG). The overall conformation of the active state CB2-G_i_ complex resembles that of the active state CB1-G_i_ complex and both ligands exhibit the same binding pose. As with the CB1, the CB2 N-termini also forms a short helix over the ligand-binding pocket. While there is an outward movement of TM6 between both receptors, CB2 undergoes minor conformational changes compared with CB1. CB2 activation results in an 11 Å outward movement of the intracellular part of TM6, allowing for G protein binding. This also results in the cytoplasmic portion of TM5 extending and moving outward by ∼6 Å to form contacts with the α5 helix of G_αi_. Notably, this differs from the active state CB1-AM841 structure which only experiences extension of TM5. Likely, the outward movement of TM5 in CB2 is due to G210^5.59^ (corresponding residue M295^5.59^ in CB1) which provides improved flexibility to the helix. Upon activation, the ionic lock between R131^3.50^ and D240^6.30^, which maintains the ground state, is broken (Figure 1B). This results in the extension of R131^3.50^ toward TM7, forming a hydrogen bond with G352 of the α5 helix. Additionally, there is a 4 Å inward displacement of the NPxxY motif (N295^7.49^, P296^7.50^, Y299^7.52^), inducing a conformational change in H8 and ICL1 allowing for a closer interaction between CB2 and the G protein.

A subsequent study captured CB2-G_i_ in complex with the agonist WIN55,212-2 (PDB: 6PT0) [41]. The overall structures of AM12033-CB2-G_i_ and WIN55,212-2-CB2-G_i_ are nearly analogous. The naphthalene moiety of WIN55,212-2 forms π–π interactions with F91^2.61^ and F94^2.64^ and hydrophobic interactions with F87^2.57^, H95^2.65^, P184^ECL2^, and F281^7.35^. The core of WIN55,212-2 forms π–π interactions with F117^3.36^ and W258^6.48^ and interacts with I110^3.29^, V113^3.32^, F183^ECL2^, V261^6.51^, and M265^6.55^. The morpholine moiety adopts a chair conformation and forms hydrophobic interactions with F183^ECL2^, I186^ECL2^, and W194^5.43^. When compared with the antagonist-bound CB2 structure, AM10257 resides deeper within the pocket by 2.8 Å, stabilizing the toggle switch W258^6.48^ in the inactive conformation.

Upon obtaining the agonist- and antagonist-bound structures of both CB1 and CB2, it was observed that the CB2 antagonist-bound structure [34] resembles the agonist-bound CB1 structure [29]. Not only are the orthosteric binding pockets similar in volume, but the arrangement of residues at the ligand-binding interface are nearly identical. Both structures also depict a short helix before Y24^N-term^ of CB2 and F108^N-term^ of CB1. Exploiting this relationship between CB1 and CB2 may allow for the development of future ligands that are antagonists at one receptor, while being an agonist at the other.

Thus far, active state cannabinoid receptor structures have been solved with non-selective agonists. The most recent CB2-G_i_ complex structures include one with LEI-102 (PDB: 8GUT), a CB2-selective agonist, alongside agonists APD371 (PDB: 8GUQ), HU308 (PDB: 8GUS), and CP55,940 (PDB: 8GUR) [42]. Although LEI-102 primarily interacts with CB2 through hydrophobic interactions, a hydrogen bond was resolved with the residue T114^3.33^. Li et al. propose that lipophilic ligands, such as HU308, may access the orthosteric pocket through the phospholipid bilayer, while polar ligands, such as LEI-102 and APD371, may enter through an alternative route.

### The G protein binding interface of CB1 and CB2

The CB1-G_i_ complex displays a similar orientation to the Rho-G_i_ structure [43] as there is an 18° rotation of the G protein when in complex with the receptor. This is thought to be due to the extended TM5 of CB1 and the extensive interactions of the N-termini of the α5 helix of G_i_. Additional sites of contact occur at ICL2, TM5, TM6, and H8 of CB1.

The interactions between the G protein and CB2 resemble those of CB1 where the primary interactions occur between the α5 helix of G_αi_ and ICL2, TM5, and TM6. Similar to CB1, there is an ∼20° rotation of the G protein compared with other GPCR-G protein structures. This is potentially due to the extension of TM5 or the additional interactions with the α5 helix, such as S222^5.71^ and H226^5.75^ of CB2 and R311^5.75^ of CB1 with D337 and Q333 of α5 helix. CB2 forms more extensive contacts with the G protein α5 helix than CB1, eight pairs of hydrogen bonds compared with the five CB1 forms. These pairs include K67^2.37^ and S69^2.39^ with D350 and C351; K142^ICL2^ with N347 and D250; R131^3.50^ with C351; H219^5.68^ and H226^5.75^ with D341 and D337; and R242^6.32^ with F254. Additionally, ICL3 in CB2 is more structured that CB1 and directly contacts G_i_ with hydrogen bonds between Q227^ICL3^ and R229^ICL3^ and Q304 and E297.

## Molecular modeling

Prior to the release of the inactive and active state cannabinoid receptor structures, computational analyses derived structural information from other GPCRs such as rhodopsin [11,44–52] and the β2-adrenergic receptor [53–55]. While the mechanism at which endocannabinoids such as 2-arachidonoylglycerol (2-AG) and N-arachidonoylethanolamide (AEA) enter the orthosteric pocket continues to be discussed, MD simulations were the first to propose a transient entry through the phospholipid bilayer [56]. Here, V351^6.43^ and I354^6.46^ of CB1 act as an AEA recognition motif and AEA proceeds to establish a hydrogen-bond with the exposed backbone of P358^6.50^ where it can then access the orthosteric pocket between TM6 and TM7. A later study confirmed a similar mechanism between 2-AG and CB2 [57]. It was these proposed interactions with TM6 that lead to the development of AM841 [58]. AM841 is a Δ^9^-THC derivative designed to covalently bind to the receptor via an isothiocyanate group. It was hypothesized that if cannabinoids were entering the receptor between TM6 and TM7, then the isothiocyanate group of AM841 would react with C6.47 more readily than other cysteine residues in the orthosteric site. In support of this hypothesis, it was found that AM841 establishes a covalent adduct with C355^6.47^ of CB1 and C257^6.47^ of CB2 [59]. However, crystallographic and cryo-EM structures exhibit a tight association between TM6 and TM7, suggesting that ligands may enter through the more flexible junction, potentially between TM1 and TM7 for example [27].

Modern MD simulations expand on the CB1 and CB2 structural data being acquired through cryo-EM and crystallography. Ji et al. [60] applied a series of computational methods to predict binding affinities and identify subtype-selective ligands. To systematically identify residues unique to active state CB1 or CB2, the authors scored the free energy of ligand-residue interactions. This method identified F170^2.57^ and S383^7.38^ as selective residues for CB1 activation while I186^ECL2^ was selective for CB2 activation. Previous mutational studies support the importance of CB1 S383^7.38^ to agonist binding, as mutating to alanine reduced the affinity for CP55,490 for the receptor compared with wild type [61].

## Allosteric modulation of CB1

Allosteric modulators can bind outside of the conical orthosteric binding site, where they can alter the conformation of the receptor, and can influence the affinity/efficacy of the orthosteric ligand. While negative allosteric modulators (NAMs) decrease the affinity/efficacy of the orthosteric ligand and positive allosteric modulators (PAMs) enhance the effects of the orthosteric ligand. The AM841- and AM11542-bound CB1 structures (PDB: 5XR8 and 5XRA) each captured a cholesterol molecule on the extrahelical surface of TM2, TM3, and TM4. Therefore, it had been proposed that cholesterol may act as an allosteric modulator at CB1. However, the FUB-CB1-G_i_ structure (PDB: 6N4B) exhibits a conformational change at F155^2.42^ and F237^4.46^, resulting in their occupation of the cholesterol-binding site. The residues implicated in cholesterol binding are not conserved between CB1 and CB2 and F4.46 is entirely unique to CB1 in Class A GPCRs.

In 2019, the first crystal structure of CB1 with the NAM ORG27569 was resolved (PDB: 6KQI) [62]. CB1 was in complex with the full, potent agonist CP55,940 occupying the orthosteric binding site and ORG27569 spanning across TM4, partially overlapping the cholesterol-binding site. Despite being bound to a potent agonist, CB1 remains in the inactive conformation, designated by the location of TM6 relative to TM3 and the conformation of the twin toggle switch. A hydrogen bound was resolved between the hydroxylpropyl group of CP55,940 and S173^3.29^, as well as van der Waals interactions between the aliphatic tail and W356^6.48^.

A later study incorporated 3′-trifluoromenthyl-phenylalanine, an unnatural amino acid, into CB1 to capture various receptor confirmations using fluorine-19 nuclear magnetic resonance spectroscopy [63]. The agonists CP55,940 and AM11542 each produced population peaks distinct from the antagonist-bound CB1. These peaks were designated as the active and inactive state receptor respectively. Interestingly, the AM841-bound CB1 showed a population split with 50% active and inactive. The addition of ORG27569 produced a separate population peak for the agonist-bound CB1; while no spectral changes to the apo or antagonist-bound CB1 occurred.

To further understand ORG27569 modulation at CB1, Wang et al. [63] set out to crystalize the CP55,940-ORG27569-CB1 complex. However, no electron density was present for ORG27569 (PDB: 7V3Z). Instead, a cholesterol molecule was identified on extrahelical surface of TM2, TM3, and TM4 of this complex, similar to other agonist-bound CB1 structures [29]. To explain the differences between their structure and the previous structure [62], the authors propose that CB1 activation states progress in equilibrium through four unique conformations: the apo state, pre-active state, active-like state, and active state. In this way, they propose that ORG27569 binds to the pre-active state receptor and promotes agonist binding [62]; subsequently, from the active-like state, ORG27569 is replaced by cholesterol, and CB1 transitions into the active state [63].

In 2022, cryo-EM and crystal structures of CB1 in complex with CP55,940 and the PAM ZCZ011 were determined [64]. The crystal structure is of CP55,940-ZCZ011-CB1 (PDB: 7FEE), while the cryo-EM structure CP55,940-ZCZ011-CB1 is bound to G_i_ (PDB: 7WV9). Similar to the CB1-ORG27569 structure, ZCZ011 binds to the TM2-TM3-TM4 surface. However, the two allosteric modulators exhibit two distinct binding poses. ZCZ011 is 7.9 Å from the orthosteric binding site, residing on the upper half of the receptor, while ORG27569 occupies more of the lower half. ZCZ011 forms hydrophobic reactions with L165^2.52^, I169^2.56^, I245^4.54^, and V249^4.58^, while the thiophene ring of ZCZ011 forms a hydrogen bound with F191^3.27^.

## Conclusions

Overall, the significant advances in structural biology have revealed many conserved mechanisms for CB1 and CB2 to activate G proteins that are not unlike structural arrangements induced by agonists at other GPCRs. A major caveat for considering the relevance of the structures obtained is the consideration of the modifications to the receptors, the compositions of the membranes/nanodiscs as well as the presence of other proteins (G proteins for example). Cryo-EM and crystallography are powerful tools for capturing snapshots of how a receptor may pose in the environment provided, but it may not capture how the receptor, fully intact and post-translationally modified, will pose when sitting in its endogenous membrane. For example, the CB1 receptor is thought to interact with a single-transmembrane spanning CB1 receptor interacting protein (CRIP1a) yet no structures of this functional complex have been reported to date [65,66]. Obtaining additional structures, using more selective agonists and modulators, while applying more physiologically relevant environments (lipid bilayer modifications, partner membrane proteins, cytoskeleton, extracellular matrix) and allowing for more movement in the presence of partners, may continue to expand what we know about how these receptors are activated. Such studies will be highly useful for future drug design, with the aim of selectively targeting not only each receptor but nuanced signaling mechanisms which may help refine therapeutic development.

## Perspectives

- CB1 and CB2 are desirable drug targets with therapeutic potential.
- Delineating between CB1 and CB2 structures may reveal key components involved in receptor modulation and function.
- Although we have gained valuable knowledge from present structures to date, future structural determination utilizing more native environments or incorporating additional protein complexes may capture more distinct conformational poses.

## Open Access

Open access for this article was enabled by the participation of University of Florida in an all-inclusive *Read & Publish* agreement with Portland Press and the Biochemical Society under a transformative agreement with Individual.

## Abbreviations

2-AG: 2-arachidonoylglycerol
AEA: N-arachidonoylethanolamide
CNS: central nervous system
ECL2: extracellular loop II
FUB: Fubinaca
MD: molecular dynamics
TM6: transmembrane helix 6
